# Lockdown, slow down: impact of the COVID-19 pandemic on physical activity—an observational study

**DOI:** 10.1136/openhrt-2021-001600

**Published:** 2021-06-04

**Authors:** Joanne Kathryn Taylor, Haarith Ndiaye, Matthew Daniels, Fozia Ahmed, Triage-HF Plus investigators

**Affiliations:** 1 Division of Informatics, Imaging and Data Sciences, The University of Manchester, Manchester, UK; 2 Manchester Heart Centre, Manchester University NHS Foundation Trust, Manchester, UK; 3 Department of Geriatric Medicine, Manchester University NHS Foundation Trust, Manchester, UK; 4 Division of Cardiovascular Sciences, University of Manchester, Manchester, UK; 5 Division of Cell Matrix Biology and Regenerative Medicine, University of Manchester, Manchester, UK

**Keywords:** pacemaker, artificial, defibrillators, implantable, COVID-19, heart failure

## Abstract

**Aims:**

In response to the COVID-19 pandemic, the UK was placed under strict lockdown measures on 23 March 2020. The aim of this study was to quantify the effects on physical activity (PA) levels using data from the prospective Triage-HF Plus Evaluation study.

**Methods:**

This study represents a cohort of adult patients with implanted cardiac devices capable of measuring activity by embedded accelerometery via a remote monitoring platform. Activity data were available for the 4 weeks pre-implementation and post implementation of ‘stay at home’ lockdown measures in the form of ‘minutes active per day’ (min/day).

**Results:**

Data were analysed for 311 patients (77.2% men, mean age 68.8, frailty 55.9%. 92.2% established heart failure (HF) diagnosis, of these 51.2% New York Heart Association II), with comorbidities representative of a real-world cohort.

Post-lockdown, a significant reduction in median PA equating to 20.8 active min/day was seen. The reduction was uniform with a slightly more pronounced drop in PA for women, but no statistically significant difference with respect to age, body mass index, frailty or device type. Activity dropped in the immediate 2-week period post-lockdown, but steadily returned thereafter. Median activity week 4 weeks post-lockdown remained significantly lower than 4 weeks pre-lockdown (p≤0.001).

**Conclusions:**

In a population of predominantly HF patients with cardiac devices, activity reduced by approximately 20 min active per day in the immediate aftermath of strict COVID-19 lockdown measures.

**Trial registration number:**

NCT04177199.

**Key questions**

**What is already known about this subject?**

- The impact of initial strict lockdown measures implemented during the COVID-19 pandemic on daily physical activity (PA) remains poorly understood. Survey data suggest a mixed response by the population, however self-reported PA can be unreliable.

**What does this study add?**

- This study takes advantage of objective remote monitoring data from implanted cardiac devices for a cohort of cardiac patients enrolled in a prospective cohort study. PA data were shown to drop by approximately 20 min/day across the cohort studied, although weekly trends suggest activity levels were returning to baseline by 3–4 weeks post-lockdown implementation.

**How might this impact on clinical practice?**

- Regular PA is integral for the maintenance of health and well-being, particularly for those with chronic diseases such as heart failure, and age-related physical frailty. Clinicians should consider the impact of reduced activity on their patient cohort when providing advice and delivering services for chronic disease groups during the pandemic.

## Background

The COVID-19 pandemic brought restrictions on the daily lives of the global population. For at least the initial phase, the UK population was advised to isolate at home, an unprecedented public health message. Although survey data suggest a mixed response by the general population,^1 2^ the effect of the ‘lockdown’ instruction on physical activity (PA) has yet to be objectively quantified. Focusing on people living with heart failure (HF), given contemporary health advice for managing stable HF in the community typically includes regular PA as part of a healthy lifestyle,^3^ any significant drop may have negative health consequences for these patients.

Modern cardiac implantable electronic devices (CIEDs) have multiple built in sensors which allow them to monitor a wide range of physiological parameters, including PA levels, continuously and automatically. When paired with home monitoring, these data can be monitored by clinical teams.^4^

## Aims

Using activity data collected from CIEDs 4 weeks before and after the imposition of strict COVID-19 lockdown measures as part of the ongoing prospective Triage-HF Plus Evaluation (Triage-HF Plus), we aim to quantify behavioural changes in activity in a cohort of CIED patients with HF.

## Methods

The Triage-HF Plus study is a prospective observational study involving three hospital sites in North West England, UK. Briefly, Triage-HF Plus is a telephone-based clinical care pathway which uses remote monitoring data from CIEDs (Medtronic Triage Heart Failure Risk Status (HFRS)) to identify patients at increased risk of decompensated HF.^5^ The aim of the study is to evaluate workforce burden and healthcare utilisation associated with the pathway. Recruitment began on 6 September 2019 and remains ongoing. Inclusion criteria include: over 18 years, HFRS compatible CIED in situ, able to comply with remote monitoring and follow-up.

Daily activity data are collected as part of the HFRS, measured by an accelerometer within the CIED, and stored as time ‘active’ per day based on an accelerometer count threshold.^6 7^ For this evaluation, activity data were available as ‘total minutes active per week’. We extracted data for the 4 weeks preceding and following the formal announcement of strict national lockdown measures in England on the evening of 23 March 2020. Prior to this announcement, life continued largely as normal until 16 March when the prime minister announced advice to ‘stop non-essential contact with others and to stop all unnecessary travel’,^8^ however, due to ‘panic buying’ and uncertainty, it is generally accepted that strict ‘lockdown’ started in the UK after 23 March. In England, government guidance was to remain at home unless required to go outdoors for a list of very limited reasons (essential work, shopping for necessities as infrequently as possible, medical or care reasons, or a short (maximum 60 min) daily period of exercise).^9 10^ This advice did not significantly change in the 4 weeks following the announcement.

Analysis was performed on Microsoft Excel 2019 and R software (V.4.0.2, 2020). Only participants with complete activity data for all 8 weeks studied were included. Statistical tests employed include paired t-test, paired Wilcoxon-test, Welsh two sample t-test (when Bartlett test significant for homogeneity of variance), analysis of covariance (ANCOVA) for multiple categorical variables, and Pearson/Spearman’s rank correlation (for continuous variables). A p value <0.05 was considered significant, where MD denotes difference in means.

## Results

At the time of data retrieval, demographic and clinical data were available for 414 patients, of which 8 weeks of complete activity data were available for 311 patients (75.1%). An overview of the studied population is shown in table 1.

**Table 1:** Demographics table

Demographics		n (%)
Hospital sites	Manchester Heart Centre	117 (37.6)
	Pennine Acute Hospitals	102 (32.8)
	Wythenshawe (Manchester)	92 (29.6)
Age (years) *Missing data n=3*	18–49	19 (6.2)
	50–59	40 (13.0)
	60–69	76 (24.7)
	70–79	121 (39.3)
	80+	52 (16.9)
Gender	Male	240 (77.2)
BMI *Missing data n=6*	<18.5	2 (0.7)
	18.5–24.9	68 (22.3)
	25–29.9	100 (32.8)
	>30	135 (44.3)
Ethnic origin *Missing data n=2*	White British	259 (83.8)
	White other	30 (9.8)
	Asian	14 (4.5)
	Black	4 (1.3)
	Mixed	1 (0.3)
	Other	1 (0.3)
Index of multiple deprivation decile* *Missing data n=10*	Median decile	4 (range 1–10)
Device type *Missing data n=1*	CRT ICD	204 (65.8)
	CRT PPM	55 (17.7)
	ICD	51 (16.5)
Smoking status *Missing data n=4*	Current smoker	30 (9.8)
	Former smoker	154 (50.2)
	Non-smoker	123 (40.1)
***Comorbidities***		
Heart failure *Missing data n=4*	HFREF	245 (79.8)
	HFPEF	34 (11.1)
	HF with unknown EF	4 (1.3)
NYHA class† *Missing data n=4*	I	72 (24.7)
	II	149 (51.2)
	III	69 (23.7)
	IV	1 (0.3)
Depression *Missing data n=7*	Yes, current depression	38 (12.5)
	Yes, previous depression	33 (10.9)
CKD 3 or greater *Missing data n=12*		126 (42.1)
Diabetes		228 (73.3)
Hypertension *Missing data n=4*		140 (45.6)
Atrial fibrillation or flutter *Missing data n=1*	Permanent/persistent	90 (29.0)
	Paroxysmal	44 (14.2)
	Previous, ablated	5 (1.6)
Ischaemic heart disease *Missing data n=8*		158 (52.1)
Chronic obstructive pulmonary disease *Missing data n=7*		36 (11.8)
Adult congenital heart disease *Missing data n=3*		8 (2.6)
Frailty *PRISMA 7 score* ≥*3*		174 (55.9)

*Where 1=most deprived area, 10=least deprived area.

†For patients with HF only.

BMI, body mass index; CKD, chronic kidney disease; CRT, cardiac resynchronisation therapy device; EF, ejection fraction; HF, heart failure; HFPEF, HF with preserved ejection fraction; HFREF, HF with reduced ejection fraction; ICD, implanted cardiac defibrillator; NYHA, New York Heart Association; PPM, pacemaker.

Of note, the majority of the studied population (77.2%) were men, with 56% over 70 years of age. Most had a high body mass index (BMI) (77% over 25). Two hundred and eighty-three (92.2%) patients had established HF, with 52.2% NYHA (New York Heart Association) class II. The majority were fitted with a cardiac resynchronisation device with defibrillator. Comorbidities included: diabetes (73.3%), ischaemic heart disease (52.1%), hypertension (45.6%), atrial fibrillation (44.6%), chronic kidney disease (42.1%) and frailty (55.9%, defined as a PRISMA-7 score ≥3).^11^

79.1% (246) of the cohort had a reduction in activity post-lockdown. Median activity per day in the 4 weeks pre-lockdown was 134.7 min/day (min/day) compared with 113.9 min/day in the 4 weeks post-lockdown. Post-lockdown there was a significant reduction in activity (p<0.001), equating to a reduction of 20.8 active min/day.

**Figure 1:**
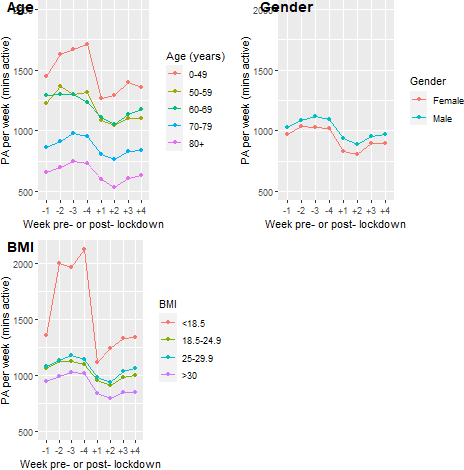
Physical activity pre-first and post-first UK lockdown by age, gender and BMI. Note only 16 participants with BMI <18.5 which may explain marked deviation in PA from other BMI groups. BMI, body mass index; PA, physical activity.

**Figure 2:**
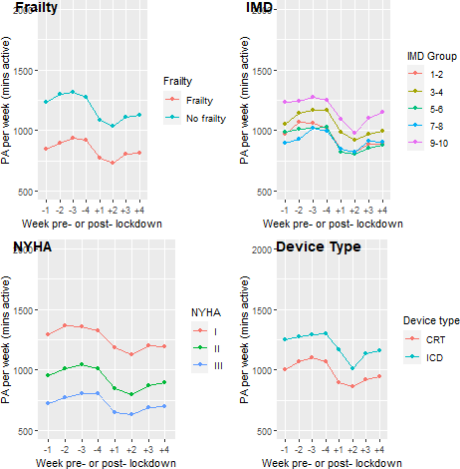
Physical activity pre-first and post-first UK lockdown by frailty, IMD, NYHA and device type. Note NYHA class IV not included as n=1. IMD, index of multiple deprivation; NYHA, New York Heart Association Functional Classification; PA, physical activity.

This reduction was uniform across the cohort (as demonstrated in figures 1 and 2), with subgroup analyses demonstrating a small but significantly more pronounced drop in activity for females (MD=6.2, 95% CI 0.1 to 12.3, p=0.04), but no statistically significant difference with respect to age (Spearman’s r=0.03, p=0.57), BMI (Spearman’s r=−0.09, p=0.11), frailty (MD=0.9, 95% CI −4.4 to 6.3, p=0.7), or device type (MD=5.5, 95% CI −13.1 to 2.1, p=0.16). A consistent pattern of activity across the 8 weeks studied emerged (see figure 1). PA was largely static leading up to lockdown implementation, followed by a precipitous drop in the 2 weeks post-lockdown. In the third and fourth week post-lockdown, there was a steady increase in observed activity, but this did not generally return to pre-lockdown levels (median activity week-1 138.6 min/day vs mean activity week-8 118.9 min/day, p≤0.001).

An exploratory analysis of the 65 (20.9%) patients whose total activity levels did not decrease post-lockdown found there was no significant difference in pre-lockdown activity (3839 median min active vs 3839.0 min active, p=0.62), or proportion of patients in the first or last quartile of pre-lockdown activity (first 29.2% vs 24.0%, last 27.7% vs 24.0%, p=0.48). There was no significant difference in demographics or comorbidities (as listed in table 1) between the two groups.

## Conclusion

This is the first study quantifying the behavioural effects of the public health lockdown instructions using a study cohort of HF CIED patients undergoing objective daily activity monitoring as part of clinical remote monitoring. Our results show that activity dropped significantly during lockdown across all groups by approximately 20 min/day, with a trend to return to prior levels of activity by week three/four post initial lockdown measures. Twenty per cent of the cohort did not reduce their levels of activity. Exploratory analysis of this subgroup did not reveal any clear distinguishing characteristics, and similar NYHA score, frailty and pre-lockdown activity levels would suggest these are patients with matched functional status. Longer follow-up is required to establish if this is a sustained picture.

While other studies have reported reduced PA levels associated with global lockdown (by way of survey, mobile phone data and wearable devices^12–15^) this is the first report using a Medicines and Healthcare products Regulatory Agency/European Medicines Agency approved implanted device in an HF population. Furthermore, the demographic data recorded as part of this clinical study allows us examine the impact of key clinical characteristics on activity—a limitation of previous reports on lockdown activity.

This study demonstrates a rapid, quantifiable, negative effect on PA regardless of age, gender, BMI or frailty status—something perhaps never seen before in this generation. Further follow-up is required to establish if this change is sustained, or whether patients will adapt to keep active by other means or return to prior levels of activity in the long-term. Multiple studies have demonstrated a positive correlation between activity and overall health, and particularly for an older population with HF and/or frailty.^16–18^ Even short periods of sedentary behaviour can result in muscle wasting and functional decline.^19^ The long-term consequences of reduced activity in this population is unclear. As we prepare for a second, and possibly recurrent waves of infection in the absence of an effective vaccine, the risk/benefit discussion surrounding stringent lockdown and shielding measures for high-risk groups should consider these implications which may be possible to mitigate with regular exercise programmes, for example.

This study was limited to a predetermined cohort with data for the 4 weeks before and after lockdown making it difficult to extrapolate and predict the long-standing, clinically relevant adverse effect of this reduction in activity. The way in which activity is captured by the device is limited in two major ways: first PA below the device threshold would not be stored by the device, and second static exercise (such as an exercise bike) would not reliably trigger the accelerometer. Although evidence suggests device accelerometery strongly correlates with activity as measured by external monitors for the majority of HF patients,^20^ these limitations are pertinent when considering patients may switch to home gym equipment in response to the restrictions.

Finally, the study participants of the Triage-HF Plus study were largely of white British ethnicity (83.8%). The generalisability of our results to the UK broader population is of interest, but beyond the scope of this report.

In conclusion, in a UK population of HF patients with compatible cardiac devices undergoing remote monitoring, PA reduced by approximately 20 min/day in the immediate aftermath of strict COVID-19 lockdown measures.

## Data Availability

All data relevant to the study are included in the article. Data supporting this article be made available to fellow researchers on request as soon as possible, wherever legally and ethically possible.

## References

[1] Robinson E , Boyland E , Chisholm A , et al . Obesity, eating behavior and physical activity during COVID-19 lockdown: a study of UK adults. Appetite 2021;156:104853. 10.1016/j.appet.2020.104853 33038479 PMC7540284

[2] Cheval B , Sivaramakrishnan H , Maltagliati S , et al . Relationships between changes in self-reported physical activity, sedentary behaviour and health during the coronavirus (COVID-19) pandemic in France and Switzerland. J Sports Sci 2021;39:1–6. 10.1080/02640414.2020.1841396 33118469

[3] Piña IL , Apstein CS , Balady GJ , et al . Exercise and heart failure. Circulation 2003;107:1210–25. 10.1161/01.CIR.0000055013.92097.40 12615804

[4] Ahmed FZ , Crosbie C , Kahn M , et al . Protecting the most vulnerable during COVID-19 and beyond: a case report on the remote management of heart failure patients with cardiac implantable electronic devices. Eur Heart J Case Rep 2020;4:1–6. 10.1093/ehjcr/ytaa249 PMC7499530 33089059

[5] Ahmed FZ , Taylor JK , Green C , et al . Triage‐HF plus: a novel device‐based remote monitoring pathway to identify worsening heart failure. ESC Heart Fail 2020;7:108–17. 10.1002/ehf2.12529 PMC7083434 31794140

[6] Conraads VM , Spruit MA , Braunschweig F , et al . Physical activity measured with implanted devices predicts patient outcome in chronic heart failure. Circ Heart Fail 2014;7:279–87. 10.1161/CIRCHEARTFAILURE.113.000883 24519908

[7] Rosman L , Lampert R , Sears SF , et al . Measuring physical activity with implanted cardiac devices: a systematic review. J Am Heart Assoc 2018;7:e008663. 10.1161/JAHA.118.008663 29773575 PMC6015387

[8] Prime Minister’s statement on coronavirus (COVID-19): 16 March 2020. Available: https://www.gov.uk/government/speeches/pm-statement-on-coronavirus-16-march-2020

[9] Prime Minister’s statement on coronavirus (COVID-19): 23 March 2020. Available: https://www.gov.uk/government/speeches/pm-address-to-the-nation-on-coronavirus-23-march-2020

[10] The Times . An hour’s walk should do, says Michael Gove, 2020. Available: https://www.thetimes.co.uk/article/an-hours-walk-should-do-says-michael-gove-9hq2qjsqb

[11] Raîche M , Hébert R , Dubois M-F . PRISMA-7: a case-finding tool to identify older adults with moderate to severe disabilities. Arch Gerontol Geriatr 2008;47:9–18. 10.1016/j.archger.2007.06.004 17723247

[12] Pépin JL , Bruno RM , Yang R-Y , et al . Wearable activity Trackers for monitoring adherence to home confinement during the COVID-19 pandemic worldwide: data aggregation and analysis. J Med Internet Res 2020;22:e19787. 10.2196/19787 32501803 PMC7307323

[13] Vinceti M , Filippini T , Rothman KJ , et al . Lockdown timing and efficacy in controlling COVID-19 using mobile phone tracking. EClinicalMedicine 2020;25:100457. 10.1016/j.eclinm.2020.100457 32838234 PMC7355328

[14] Smith L , Jacob L , Butler L , et al . Prevalence and correlates of physical activity in a sample of UK adults observing social distancing during the COVID-19 pandemic. BMJ Open Sport Exerc Med 2020;6:e000850. 10.1136/bmjsem-2020-000850 PMC7358093 34192006

[15] Moore SA , Faulkner G , Rhodes RE , et al . Impact of the COVID-19 virus outbreak on movement and play behaviours of Canadian children and youth: a national survey. Int J Behav Nutr Phys Act 2020;17:1–11. 10.1186/s12966-020-00987-8 32631350 PMC7336091

[16] Rogers NT , Marshall A , Roberts CH , et al . Physical activity and trajectories of frailty among older adults: evidence from the English longitudinal study of ageing. PLoS One 2017;12:e0170878. 10.1371/journal.pone.0170878 28152084 PMC5289530

[17] McNaughton SA , Crawford D , Ball K , et al . Understanding determinants of nutrition, physical activity and quality of life among older adults: the wellbeing, eating and exercise for a long life (WELL) study. Health Qual Life Outcomes 2012;10:109. 10.1186/1477-7525-10-109 22966959 PMC3479030

[18] Springer J , Springer J-I , Anker SD . Muscle wasting and sarcopenia in heart failure and beyond: update 2017. ESC Heart Fail 2017;4:492–8. 10.1002/ehf2.12237 29154428 PMC5695190

[19] Welch C , K Hassan-Smith Z , A Greig C , et al . Acute Sarcopenia Secondary to Hospitalisation - An Emerging Condition Affecting Older Adults. Aging Dis 2018;9:151–64. 10.14336/AD.2017.0315 29392090 PMC5772853

[20] Pressler A , Danner M , Esefeld K , et al . Validity of cardiac implantable electronic devices in assessing daily physical activity. Int J Cardiol 2013;168:1127–30. 10.1016/j.ijcard.2012.11.050 23201084

